# Supplementary material for: Magnetically-driven phase transformation strengthening in high entropy alloys
Source: Nat Commun. 2018 Apr 10;9:1363. doi: 10.1038/s41467-018-03846-0 (PMC5893566; doi:10.1038/s41467-018-03846-0)
Supplement: Supplementary file 1 — Supplementary Information [file 41467_2018_3846_MOESM1_ESM.pdf]

SUPPLEMENTARY FIGURES:

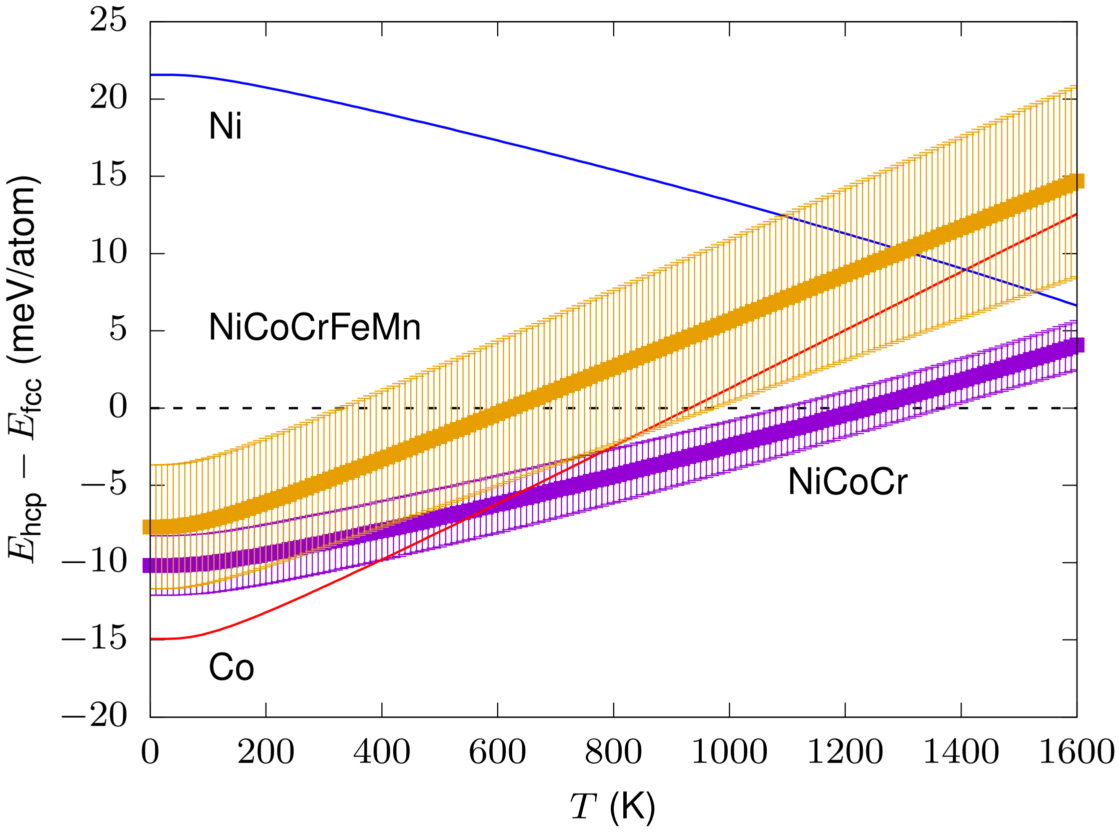

Supplementary Figure 1. Free energy difference between hcp and fcc phases of CrMnFeCoNi, computed with DFT and the harmonic approximation for phonon calculations. The relative energies of CrCoNi, Co and Ni are computed within the quasi harmonic approximation and are repeated from Fig 2 in the main text.

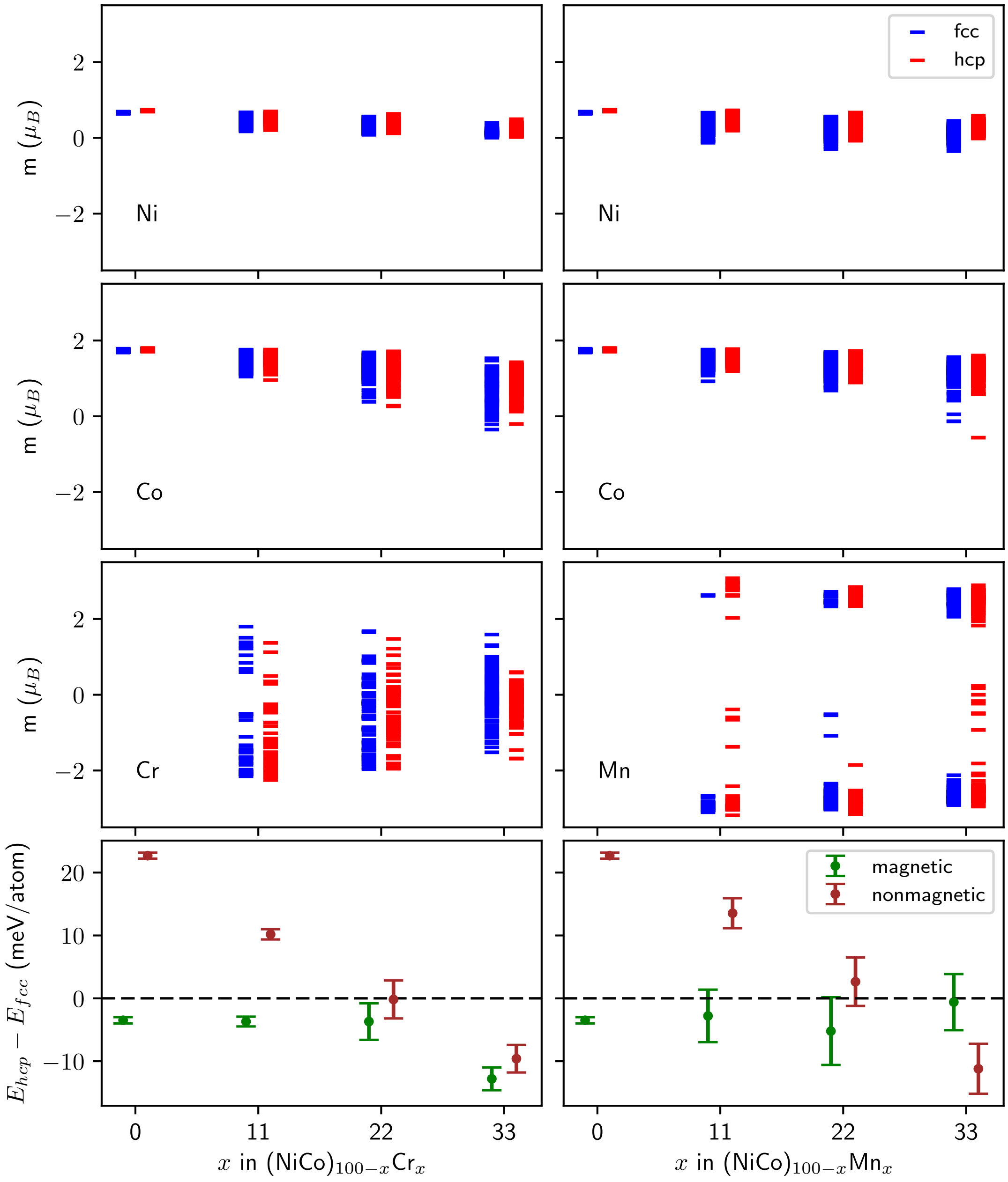

Supplementary Figure 2. Atomic magnetic moments and free energy differences between hcp and fcc phases of CrCoNi and MnCoNi as a function of Cr/Mn concentration. The top three rows showatomic magnetic moments distribution of three components in the fcc and hcp phases. The last row shows energy gap between hcp and fcc phases, with spin polarization on and off. Each energy point includes 6 individual calculations, while the error bars show the energy scatter among the six energies for each structure.

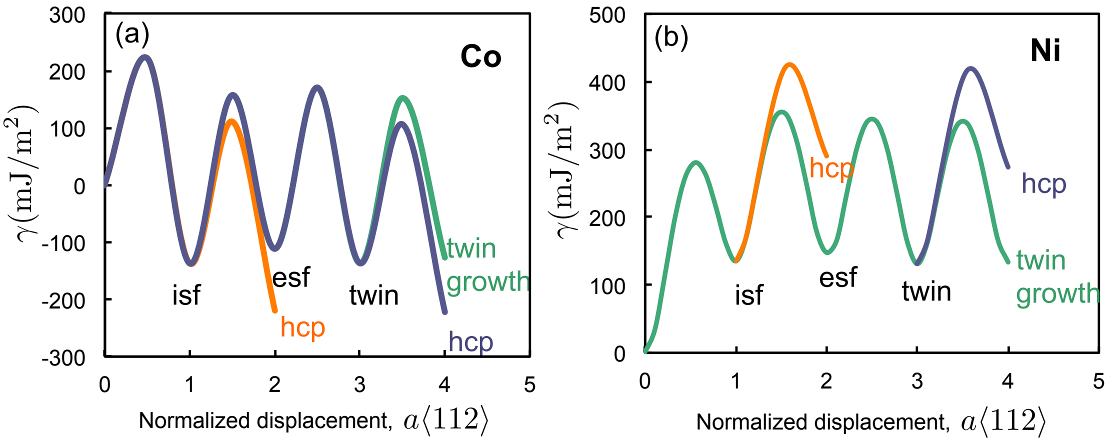

Supplementary Figure 3. *Ab initio* generalized planar fault energies versus normalized shear displacements on successive {111} planes in (a) fcc Co and (b) Ni. The energy pathways for fcc Co show negative isf and favorable hcp formation similar the average response of CrCoNi shown in Figure 4.

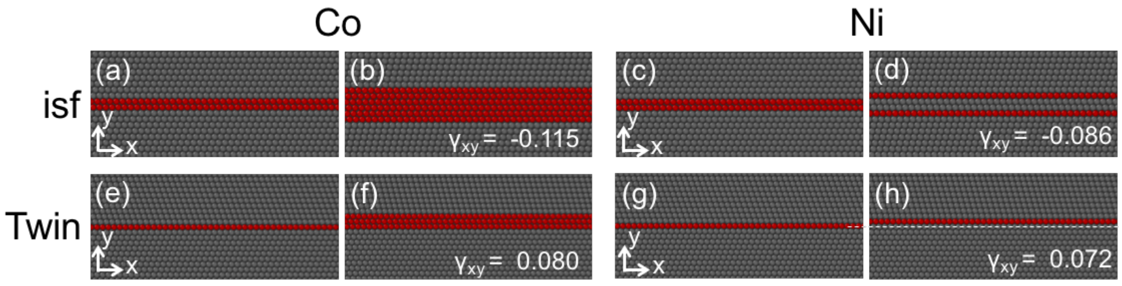

Supplementary Figure 4. Atomistic modeling of the response of an extended isf and a twin boundary to resolved shear strain, acting on the y plane in x direction, in fcc Co and Ni. The isf in Co (a) and Ni (c) turns into a hcp layer (b) and esf (d) respectively. The twin boundary in Co (e) also turns into a hcp region (f) while the Ni twin boundary (g) grows by a layer (h) under sufficient resolved shear strain. The initial position of the twin boundary is shown by a dashed line in (h) for comparison. The minimum corresponding shear strains for each transformation are also shown.

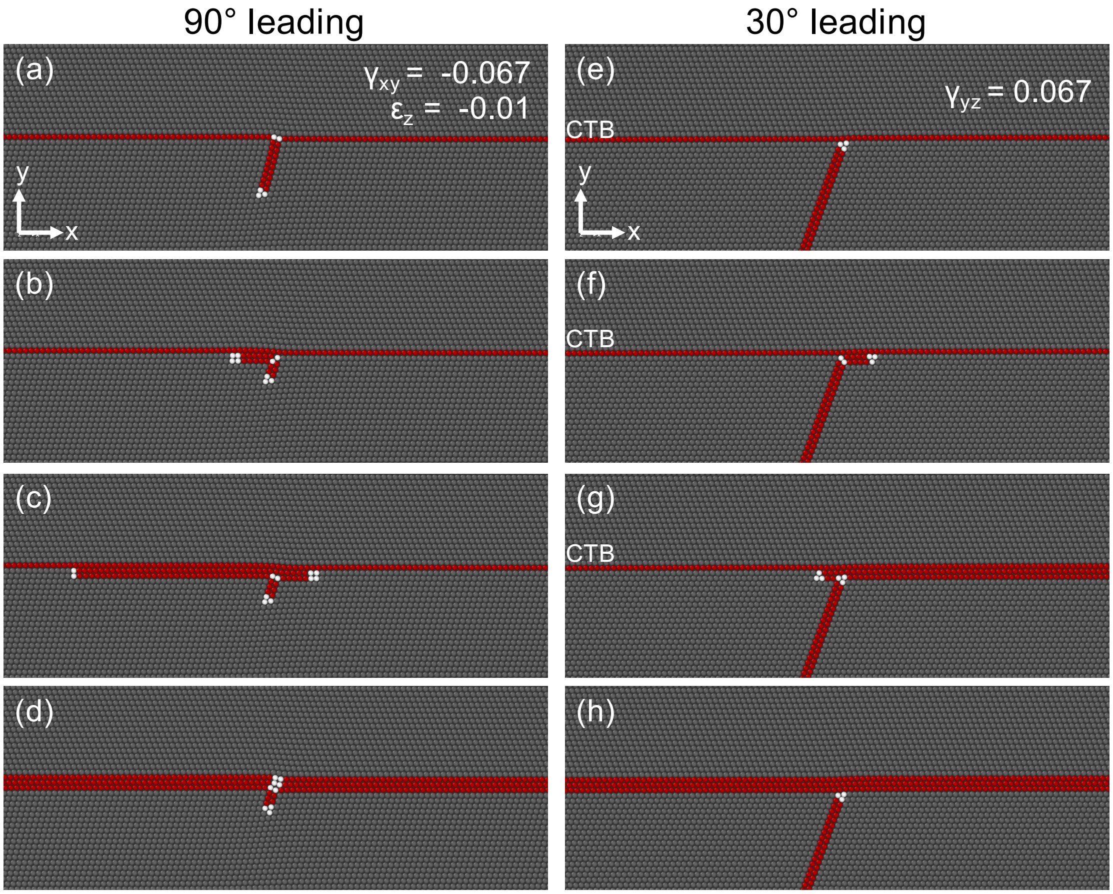

Supplementary Figure 5. Heterogeneous formation of hcp lamella from interaction of mixed 60 ° dislocations with a twin boundary in fcc Co. (a)-(d) show different stages of the interaction when the 90 ° partial dislocation is leading and (e)-(h) show the same where the 30 ° partial dislocation is leading. In both cases the leading partial gets trapped by the boundary and acts as a source of stress concentration, nucleating new Shockley partials on the twinning plane that transforms the twin boundary into a hcp lamella.

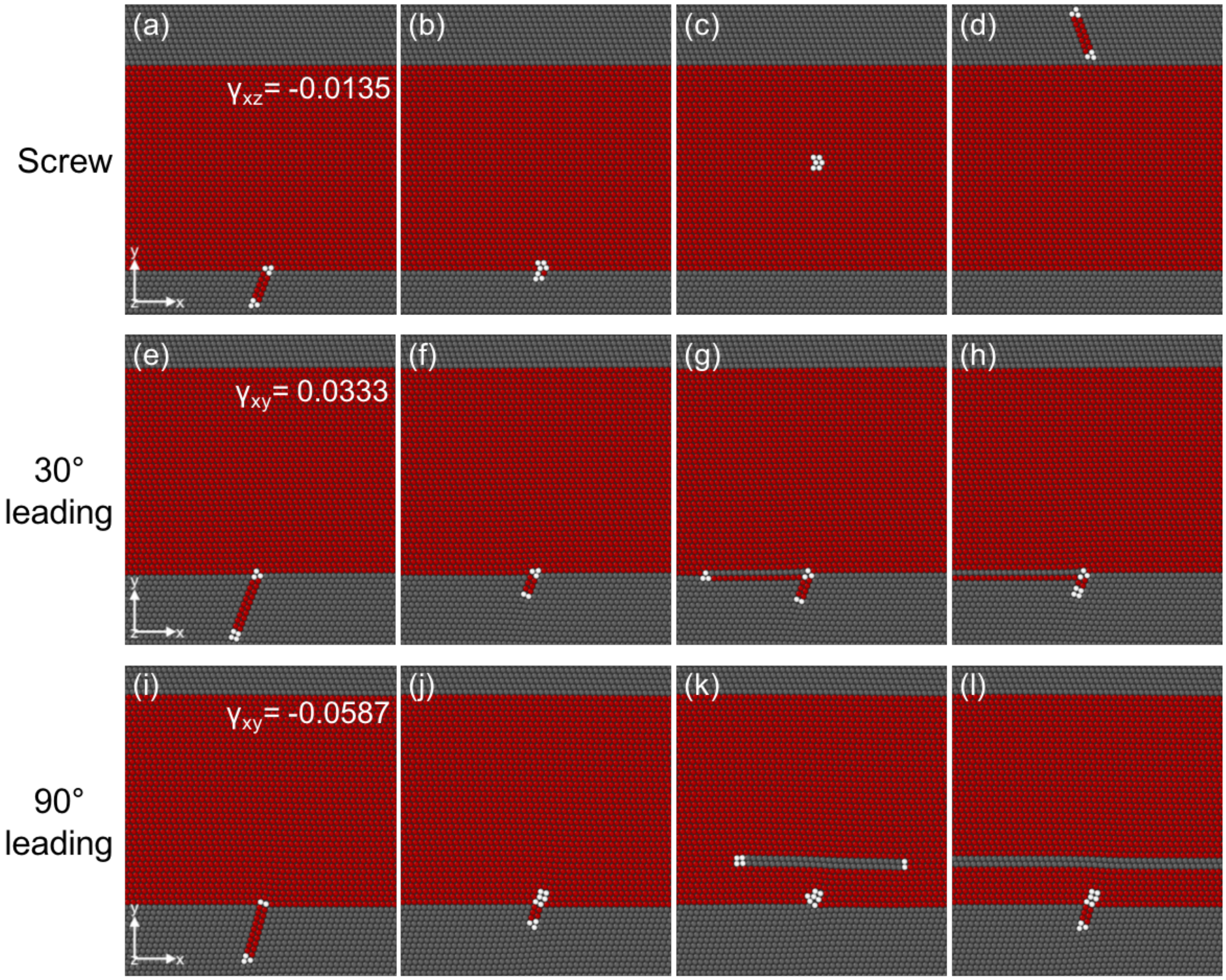

Supplementary Figure 6. Interaction of a well-developed hcp region inside the fcc Co with incoming screw (a)-(d), and mixed dislocations. In case of the mixed dislocation, (e)-(h) and (i)-(l) correspond to the the 30° and 90° leading partial dislocations. In all cases the leading partial is trapped at the interface. The screw dislocation passes through the hcp layer and completely transfers to the fcc region on the other side under sufficiently large strain. In the case of mixed dislocations, the trapped dislocations at the interface facilitate the nucleation of new Shockley partials that can locally recover fcc stacking, but effectively block transmission through the hcp layer. See Videos S1 for the interaction of the hcp region with the incoming screw dislocation, and for the mixed dislocation, Videos S2 and S3 for the 30° and 90° leading partials, respectively.

SUPPLEMENTARY TABLES:

Supplementary Table 1. Properties of Co and Ni in hcp and fcc phases computed with the Co-Ni EAM potential in comparison with DFT calculations. The significant difference between the EAM and DFT planar fault energies of fcc Co can be explained by the fact that the $E_{hcp} - E_{fcc}$ predictions of EAM and DFT differ by 4 meV/Å, but is negative in both cases.

Properties	DFT		EAM	
	Co	*Ni*	*Co*	*Ni*
hcp energy/atom (eV/atom)	−7.112	−5.5499	−4.3910	−4.4279
fcc energy/atom (eV/atom)	−7.0938	−5.5725	−4.3849	−4.4500
hcp lattice constant a (Å)	2.4947	2.4890	2.5187	2.4819
hcp lattice constant c (Å)	4.0283	4.0804	4.0557	4.1048
fcc lattice constant (Å)	3.5218	3.5211	3.5642	3.5200
isf energy (mJ/m^2)	−137.33	135.362	−32.84	134.67
esf energy (mJ/m^2)	−111.5	147.84	−29.27	135.84
twin boundary energy (mJ/m^2)	−63.45	65.66	−14.68	67.81

SUPPLEMENTARY NOTE I: FREE ENERGY CALCULATIONS OF THE QUINARY CrMnFeCoNi HIGH ENTROPY ALLOY

Vibrational entropic contributions to the free energy are calculated from the phonon spectra, obtained from the finite displacement method and DFT forces[1]. The harmonic approximation assumes small atomic displacements around their equilibrium position. The potential energy then can be expanded as a function of atomic displacements, with coefficients designating the zeroth, first, second and higher order force constants. In the limit of small displacements at constant volume, the potential energy is considered to the second order term as the harmonic approximation. The second order force constants are then computed by displacing individual atoms and computing the forces on other atoms. Applying the crystal symmetry often reduces the number of necessary independent finite displacements. In case of the high entropy alloys, similar displacement vectors are not necessarily equivalent, due to varying types of nearest neighbors.

Based on the finite displacement method, harmonic approximation (HA) calculations of a 60-atom structure of the CrMnFeCoNi alloy requires $60\times6=360$ static VASP calculations to calculate the force constants. We find the electronic convergence criterion must be between 1E-6 to 1E-8 eV (depending on the specific structure) to obtain real and positive frequencies for all phonons. In addition, all of the 360 static calculations must have universal VASP settings to minimize system errors, otherwise imaginary parts appear in the phonon band structures.

However, in case of the quinary high entropy alloy, it is not always possible to achieve convergence for all 360 VASP static calculations with the same settings, even within 200–300 electronic steps. Specifying the initial magnetic moments for every atom based on the highly relaxed structure without displacement greatly helps the electronic convergence issue, but doesn't always work. We have tested VASP mixing parameters including ALGO, AMIX, BMIX, AMIX_MAG, BMIX_MAG, as well as starting from a previously converged charge density file. Even with all these parameters carefully tuned, it is not always possible to finish an individual HA calculation. We believe this convergence issue is due to the complicated magnetic structure of this alloy.

Quasi-harmonic approximations (QHA) considers volume dependence of phonon frequencies to approximate anharmonic effect. To perform a QHA calculation, we need to execute

HA calculations on the same structure but with a series of different volumes. All these HA calculations of the same structure must be successfully finished. However, this is extremely difficult for the quinary alloy, given that convergence is already a big issue in any HA. We performed such calculations with no difficulty for the ternary CrCoNi alloy as presented in Fig.2 of the main text.

To have a baseline for comparison, and in light of the convergence issue encountered in obtaining accurate force constants in the CrMnFeCoNi, we only used HA to compute the phase stability of the quinary HEA. Supercells consisting of 60 atoms are used, with both fcc and hcp structures and a gamma-centered k-mesh of $2 \times 6 \times 2$. The supercells are relaxed with an electronic energy convergence criterion of 10E-6 eV. Both the lattice vectors and atomic coordinates are relaxed until the forces on atoms are smaller than 10 meV/Å. The finite displacement calculations use electronic criterion of 10E-6 eV, which is decreased to10E-8 eV in cases where such accuracy was needed to eliminate the imaginary components in the phonon band structures. We verified that the two values generate almost identical free energy values, as long as the phonon band structure has no imaginary parts.

Supplementary Figure 1 compares the relative hcp-fcc phase energies of CrMnFeCoNi with those of CrCoNi, Co and Ni, repeated from Fig.2 in the main text. This figure should be interpreted with caution. First, as noted above the quinary alloy free energies are computed with HA while the other cases are performed with QHA. Second, the error bars corresponding to the quinary alloy energy values are significantly larger compared to the ternary alloy. This is due to the limited number of converged configurations (only 5 fcc and 3 hcp structures). More configurations are needed to increase the reliability of predictions for this alloy, but obtaining such configurations poses a great practical challenge as explained above. Nevertheless, the average behavior of the two alloys follow the same trend, but the deviation from average is much more significant in the quinary alloy. Also, considering the various approximations in obtaining the hcp-fcc transition temperature, this transition still seems to occur at much lower temperatures for the quinary alloy compared to the ternary CrCoNi.

SUPPLEMENTARY NOTE II: COMPARING THE MAGNETIC BEHAVIOR OF Cr AND Mn

Supplementary Figure 2 provides evidence for the different magnetic natures of Cr and Mn, two antiferromagnetic elements in pure form. Ternary $(NiCo)_{100\text{-}x}Cr_x$ and $(NiCo)_{100\text{-}x}Mn_x$ alloys are considered, with x being the atomic concentration of Cr or Mn. The first three rows compare the atomic magnetic moments in the fcc or hcp phase of all these alloys. Cr magnetic moments span a range of positive and negative values at all concentrations. It is noteworthy that the scatter in Cr magnetic moments decreases in the hcp phase of the equiatomic CrCoNi alloy. Overall, Cr magnetic moments seem to be strongly affected by the magnetic moments of its neighbors. On the other hand, Mn moments follow a rather bimodal distribution, fluctuating between approximately 3 and -3 μ_B regardless of the concentration. Therefore, the magnetic moments of Mn atoms do not seem to be affected by their environment. Some scatter is observed in the hcp phase of the equiatomic MnCoNi alloy but the magnetic moments for most Mn atoms are still either +3 or -3 μ_B.

Computational details are as follows. Twenty 54-atom SQS models are used: six (27+27) binary fcc and hcp, six (6+24+24) ternary fcc and hcp, six (12+21+21) ternary fcc and hcp, and two equiatomic 54-atom supercells the same as those used in Figure 4 in the main text. We considered, in total, 3 SQS for each target structure to make sure there are 6 permutations for each structure. All these SQS models have the same shape, consisting of six (111) layers with 3x3=9 atoms on each layer. The same gamma-centered k-mesh of $4 \times 4 \times 2$ is applied for all models. Electronic convergence energy is 10 E-6 eV. Lattice vectors and atomic positions are relaxed until the atomic forces are smaller than 10 meV/Å.

SUPPLEMENTARY NOTE III: FCC Co AS A SURROGATE FOR MODELING NiCoCr

Both NiCoCr and pure fcc Co have negative stacking fault energies and a more favorable hcp phase at $T = 0$K (c.f. Figures 2 and 4 (b)). Supplementary Figure 3 shows the DFT-computed energy pathways corresponding to the glide of $b = 1/6\langle 112\rangle$ Shockley partials on successive $\{111\}$ planes for (a) metastable fcc Co and (b) Ni, respectively. In examining the energy pathways for fcc Co, we reach the same conclusions previously drawn for NiCoCr:

(1) the creation of an isf is energetically favorable, but imposes a significant positive energy barrier, and (2) hcp transformation is favorable to twin formation. This trend is contrary to that observed by the energy pathways in Ni, where the ground state is the fcc structure. In the absence of a reliable interatomic potential for the Ni-Co-Cr system, fcc Co– described by an EAM potential for the Co-Ni system [4]– is used as a surrogate system to study deformation mechanisms in NiCoCr qualitatively.

Supplementary Note III.A: Homogeneous nucleation of hcp laths :

Supplementary Figure 4 compares the response of existing isf and twin boundaries in fcc Co and Ni to applied shear. In fcc Co, shearing the isf (a) and twin boundary (e) causes creation of hcp regions (b) and (f), respectively. In Ni, the isf (c) and twin boundary (g) lead to the formation of an esf (d) and twin growth (h) in response to resolved shear strain. These results agree with the predictions of the energy pathway calculations.

Supplementary Note III.B: Heterogeneous nucleation of hcp laths :

The hcp lamella can also form heterogeneously, as a result of dislocation/boundary interactions. Supplementary Figure 5 shows the interaction of a 60° mixed dislocation with an existing twin boundary, forming a 3-layer hcp structure. Because of the negative stacking fault energy, the dislocation readily dissociates into 90° and 30° partials with an extended stacking fault in between. With either partial as "Leading" the interaction with the twin boundary, an hcp layer is created. Furthermore, this process can repeat over and over as the "leading" partial is recreated after each hcp thickening process. Increasing strain beyond this point does not pass the trailing dislocation through the boundary, instead, the partials that are traveling on the twin boundary will reach the fixed boundaries of the simulation, and nucleate more partials at the fixed boundaries, which keep the hcp layer growing. We did not include those snapshots, to avoid mixing artifacts of the fixed boundary conditions with actual process that result in the initial nucleation.

Supplementary Note III.C: Dislocation interaction with an existing hcp lath :

Supplementary Figure 6 shows the interaction of a well-developed hcp region containing a twin boundary with incoming screw (a)-(d) and 60° mixed dislocations. In all cases the leading partial is trapped at the interface. The screw dislocation passes through the hcp layer and completely transfers to the fcc region on the other side under sufficiently large strain. In the case of mixed dislocations, the trapped dislocations at the interface facilitate the nucleation of new Shockley partials that can locally recover fcc stacking, but effectively block transmission through the hcp layer.

SUPPLEMENTARY METHODS:

Molecular statics simulations are performed using the LAMMPS package [2] and the results are visualized using Ovito [3]. An EAM potential for the Co-Ni system [4] is used. The pure Co potential, [5] used in the development of the binary Co-Ni potential, reliably reproduces many of the basic physical properties including lattice constants, elastic constants, and cohesive energy of both fcc and hcp phases with nearly equal accuracy. Furthermore, Table S1 shows that the hcp is the more favorable phase in Co at $T = 0$K by more than 6 meV/atom, which is within 4 meV/atom of the DFT calculations for NiCoCr shown in Figure 2.

For all simulation cells, the dimensions in the x- and y-directions in terms of the fcc lattice constants for Co ($a_0 = 3.5642$ Å) [5] and Ni ($a_0 = 3.52$ Å) [6] and are approximately $100\sqrt{6}a_0$ and $120\sqrt{3}a_0$, respectively. Fixed boundary conditions are applied along the x- and y-directions, and the positions of atoms within 10 Å of these boundaries were fixed to mitigate the effects of free surfaces. Periodic boundary conditions are applied along the dislocation line in the z-direction, with a periodic distance of approximately $\sqrt{2}/2a_0$ Å. External engineering strain components are applied by changing the dimensions of the simulation cell and remapping the atomic positions.

For simulations of dislocation/boundary interactions, a dislocation is created within the fcc matrix. To prevent the dislocation from dissociating across the entire length of the grain due to the negative stacking fault energy, the dislocation is only partially relaxed. The three types of boundaries examined are a twin boundary, an intrinsic stacking fault, and an

hcp/fcc interface. The simulation cell containing the hcp/fcc interface consists of fcc matrix, approximately 40 layers of hcp, and fcc twin. For each boundary, three types of dislocation interaction are examined: screw dislocations and 60° mixed dislocations (both 30° and 90° leading).

SUPPLEMENTARY REFERENCES:

[1] Togo, A. and Tanaka, I. First principles phonon calculations in materials science. *Scripta Mater.* **108**, 1-5 (2015)

[2] Plimpton, S. Fast Parallel Algorithms for Short-Range Molecular Dynamics. *J. Comp. Phys.* **117**, 1-19 (1995)

[3] A. Stukowski, Visualization and analysis of atomistic simulation data with OVITO - the Open Visualization Tool. *Modelling Simul. Mater. Sci. Eng.* **18**, 015012 (2010)

[4] Purja Pun, G. P., Yamakov, V., Mishin, Y. Interatomic potential for the ternary Ni-Al-Co system and application to atomistic modeling of the B2-L1 0 martensitic transformation. *Modelling Simul. Mater. Sci. Eng.* **23**, 065006 (2015)

[5] Purja Pun, G.P., Mishin, Y. Embedded-atom potential for hcp and fcc cobalt. *Phys. Rev. B* **86**, 134116 (2012)

[6] Mishin, Y. Atomistic modeling of the γ- and γ'-phases of the Ni-Al system. *Acta Mater.* **52**, 1451-1467 (2004)
