# Supplementary material for: Magnetically-driven phase transformation strengthening in high entropy alloys
Source: Nat Commun. 2018 Apr 10;9:1363. doi: 10.1038/s41467-018-03846-0 (PMC5893566; doi:10.1038/s41467-018-03846-0)
Supplement: Supplementary file 3 — Description of Additional Supplementary Files [file 41467_2018_3846_MOESM3_ESM.pdf]

Description of Additional Supplementary Files

File Name: Supplementary Movie 1
Description: Interaction of the screw dislocation with the fcc/hcp boundary, shown in Supplementary Figure 4 (a)-(d). Under sufficient strain, the screw dislocation penetrates the hcp layer, glides on the hcp prism planes, and transfers to the fcc twin region on the other side.

File Name: Supplementary Movie 2
Description: Interaction of the 30◦ leading partial of the mixed dislocation with the fcc/hcp boundary, shown in Supplementary Figure 4 (e)-(h). As a result of the local stress concentration from the blockage of the leading partial, a new partial dislocation is nucleated, which glides parallel to the twin plane; this new partial transforms the hcp layer at the boundary to fcc stacking, while simultaneously transforming the below fcc layer to hcp.

File Name: Supplementary Movie 3
Description: Interaction of the 90◦ leading partial of the mixed dislocation with the fcc/hcp boundary, shown in Supplementary Figure 4 (i)-(l). Under sufficient strain, the leading 90◦ partial is effectively blocked from penetrating more than the first couple atomic layers of the hcp region. The trailing 30◦ partial pushes past the leading partial, nucleates a new partial that glides parallel to the twin plane within the hcp region, and is re-emitted into the fcc matrix.

File Name: Supplementary Movie 4
Description: Interaction of two screw dislocations with hcp/fcc region seen in HAADF images, shown in Figure 7 of the main text. A simulation cell containing regions of hcp lamella interspersed with single FCC atomic layers is constructed to resemble those observed in the HAADF image shown in Figure 5 (a). Similar structures to this are also seen during simulations in which higher strains than those shown in Figure S2 (e) are applied to an existing twin boundary in fcc Co. Under sufficient strain, both leading partials move towards the isf. When a single screw dislocation interacts with the hcp/fcc region, the leading partial is blocked. Under sufficient strain, the isf separates at the site of the dislocation interaction, and the hcp is transformed to fcc. However, in the presence of the stress field of the second screw dislocation, the interaction of the first screw dislocation results in the nucleation of a new partial that glides parallel to the isf plane while transforming one atomic layer from hcp to fcc, and the layer below that from fcc to hcp.
